# Vicarious conditioned fear acquisition and extinction in child–parent dyads

**DOI:** 10.1038/s41598-020-74170-1

**Published:** 2020-10-13

**Authors:** Marie-France Marin, Alexe Bilodeau-Houle, Simon Morand-Beaulieu, Alexandra Brouillard, Ryan J. Herringa, Mohammed R. Milad

**Affiliations:** 1 Department of Psychology, Université du Québec à Montréal, 100 Sherbrooke West Street, Montreal, QC H2X 3P2 Canada; 2 Research Center of the Institut universitaire en santé mentale de Montréal, 7331 Hochelaga Street, Montreal, QC H1N 3V2 Canada; 3 Department of Psychology, Université de Montréal, 2900 Edouard-Montpetit Blvd, Montreal, QC H3T 1J4 Canada; 4 Department of Neurosciences, Université de Montréal, 2900 Edouard-Montpetit Blvd, Montréal, QC H3T 1J4 Canada; 5 Department of Psychiatry, School of Medicine and Public Health, University of Wisconsin, 750 Highland Ave, Madison, WI 53726 USA; 6 Department of Psychiatry, New York University Grossman School of Medicine, 530 1st Ave, New York, NY 10016 USA; 7 Present Address: Child Study Center, Yale University School of Medicine, 230 S Frontage Rd, New Haven, CT 06519 USA

**Keywords:** Extinction, Fear conditioning, Human behaviour

## Abstract

The biological mechanisms involved in fear transmission within families have been scarcely investigated in humans. Here we studied (1) how children acquired conditioned fear from observing their parent, or a stranger, being exposed to a fear conditioning paradigm, and (2) the subsequent fear extinction process in these children. Eighty-three child-parent dyads were recruited. The parent was filmed while undergoing a conditioning procedure where one cue was paired with a shock (CS + Parent) and one was not (CS −). Children (8 to 12 years old) watched this video and a video of an adult stranger who underwent conditioning with a different cue reinforced (CS + Stranger). Children were then exposed to all cues (no shocks were delivered) while skin conductance responses (SCR) were recorded. Children exhibited higher SCR to the CS + Parent and CS + Stranger relative to the CS −. Physiological synchronization between the child’s SCR during observational learning and the parent’s SCR during the actual process of fear conditioning predicted higher SCR for the child to the CS + Parent. Our data suggest that children acquire fear vicariously and this can be measured physiologically. These data lay the foundation to examine observational fear learning mechanisms that might contribute to fear and anxiety disorders transmission in clinically affected families.

## Introduction

How do children learn to fear? While there have been some interesting studies to suggest that some fears are biologically inherited, other fears are most likely acquired through observation. Using sophisticated methods, recent animal studies have provided evidence for neurobiological mechanisms underlying both types of transmission. For example, animal experiments have demonstrated that a parent’s experience (e.g., odor fear conditioning) prior to conception could influence the offspring’s responses and neuroanatomy in a cue-specific manner, highlighting the biological inheritance of fears, most likely through epigenetic mechanisms^1^. Debiec and Sullivan^2^ have elegantly demonstrated that a conditioned fear to an odor expressed by a mother in the presence of the pups can be passed on to them. The authors have also shown that this phenomenon is mediated by the amygdala, a brain region essential for fear learning and expression, and that pharmacologically inactivating the pups’ amygdala prevents this fear transmission^2^. These findings illustrate how emotional trauma could be transmitted across generations and highlight the various mechanisms through which this transmission of fear occurs.

Humans are no exception to this phenomenon of intergenerational transmission of fear. More often than not, children acquire conditioned fear responses without being directly exposed to the aversive stimulation. This phenomenon is known as observational fear learning^3^. Parents transmit a lot of information to their children through their body language and their reactions to environmental stimuli. In the specific case of fear, there is some support that transmission can occur vicariously, notably through interactions between the child and the parent. Research protocols have been designed to reproduce this phenomenon in the laboratory. In these protocols, the demonstrator’s unconditioned response (e.g., frowning) serves as the unconditioned stimulus for the observer^4^. Vicarious learning has been documented across species^5^ and is particularly relevant in parent–child dyads. A recent study tested whether self-reported fear levels of children could be modulated by pairing a novel animal with an image of a facial expression (happy vs. fearful) exhibited by their mother (or an unfamiliar adult, i.e., stranger condition)^6^. Results showed that children were more afraid of the animals that were paired with the fearful faces. Fear levels were similar when the child was learning from the mother or from the unfamiliar adult^6^.

The biological correlates of observational fear learning in children remain understudied despite the fact that dysregulated biological responses to fear have been demonstrated in children^7^. It was not until recently that physiological measures were used to study vicarious fear learning in children^8,9^. However, to the best of our knowledge, fear transmission from parents to children has never been studied with physiological measures in humans. Moreover, it remains to be determined whether a fear memory that has been acquired vicariously undergoes extinction learning and whether this extinction process persists over time. Such processes are important to study, given that they reflect some key components of emotion regulation abilities^10^.

The study of vicarious fear acquisition in children is highly relevant clinically. Childhood is a key developmental period where the emergence of anxiety disorders is pronounced, with a median age of onset of 11 years old^11^. Childhood is also a time when various conditioned fears are acquired^12–14^. Since fear is at the core of anxiety disorders, it is important to examine fear development in children. The familial environment is a modulator for children’s expression and regulation of emotions, especially fear. In fact, parents’ emotional reactions can amplify or attenuate their own child’s fear responses^15,16^. It has been suggested that the parent–child dyad, rather than the child alone, should be considered as a key unit of analysis when studying emotion development^17,18^. Given that risk for anxiety and fear-based disorders is transmitted within families^19–22^, it is essential to study how children form fearful associations when learning from their parent’s experience.

**Figure 1:**
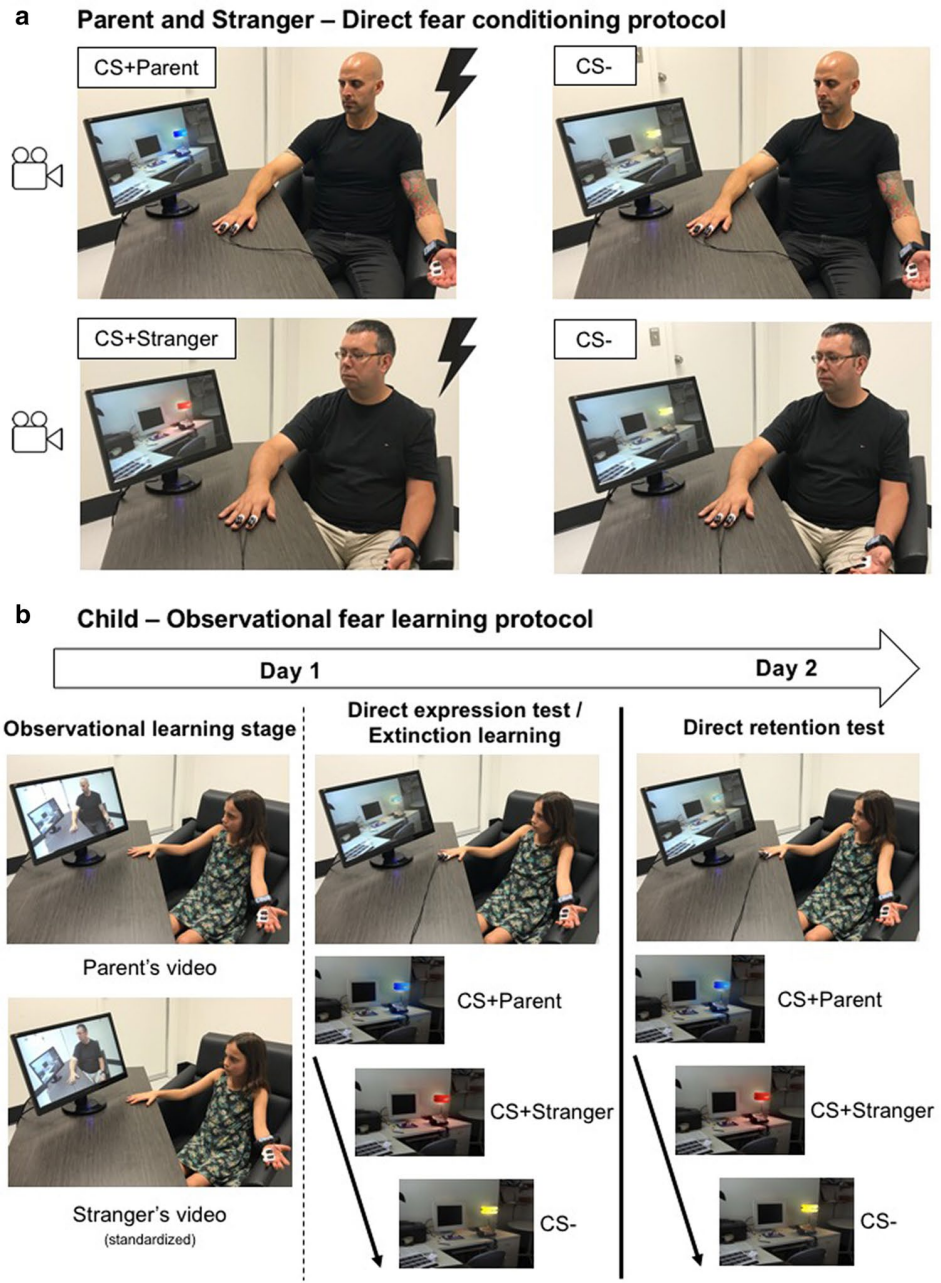
Summary of the protocol. (**a**) Parent & Stranger: Direct fear learning protocol. On Day 1, parents were exposed to a direct fear conditioning protocol where two colored lamps were presented, one was paired with a shock 5 times out of 8 presentations (CS + Parent; red or blue) while the other was never paired with a shock (CS −; yellow). A stranger adult was exposed to a similar procedure except that a different color (e.g., red) was used for the CS + . The same color that was used for the CS − of the parent was used for the stranger (yellow, CS −). Both the stranger and the adult were filmed during this procedure. Note that the stranger video was matched to the sex of the participating parent. All children participating with their mother saw the same stranger video (stranger woman) and all children participating with their father saw the same stranger video (stranger men). (**b**) Child – Observational learning stage. The child was presented with both videos (observational fear learning phase). After that, the shock electrodes were attached to the child’s hand and he/she was instructed that shocks could be delivered (no shock was delivered to the child). The child was directly exposed to all three colored lights (CS + Parent, CS + Stranger, and CS −) (direct expression test and extinction learning). On Day 2, the child was once again presented with all three stimuli (CS + Parent, CS + Stranger, and CS −) and no shock was delivered (direct retention test).

Despite the importance of observational fear learning during childhood, most studies on fear learning expose children to direct fear conditioning protocols, i.e., when the individual is directly experiencing the unconditioned stimulus. To begin to fill a knowledge gap in the literature within this domain, in the present study, we developed an observational fear learning paradigm. In this paradigm, the child observed his/her parent and an adult stranger undergoing a direct fear conditioning paradigm. Following the observation phase, the child was directly exposed to the conditioned stimuli for which the parent and the stranger were conditioned to assess whether the fear memory has been acquired (direct expression test). Given that no shocks were delivered to the child, we were also able to assess whether the fear memory would undergo extinction learning. We then tested retention of fear on the following day (Fig. 1). Finally, in order to better understand the mechanism underlying such learning and the inter-dyadic differences, we tested whether the parent–child physiological synchrony during the observational learning phase predicted the child’s physiological reaction when later exposed to the conditioned stimuli (direct expression test). We hypothesized that children would exhibit higher fear levels, as assessed by skin conductance responses (SCR), to the cues for which the parent and the stranger received shocks (CS + Parent, CS + Stranger) relative to the safety cue (CS −). We did not have specific hypotheses for extinction learning and recall, as these have not been tested in children in the context of an observational fear learning protocol.

## Results

Socio-demographics are presented in Table 1. Among the 83 dyads included in the study, three were removed from analyses because of poor data quality. Also, one child dropped out of the experiment prior to the direct expression test. Therefore, observational fear learning was tested on 80 children, whereas direct expression, fear extinction learning, and direct retention (Day 2) were tested on 79 children. Fear conditioning analyses were conducted on 72 parents, since one parent’s electrodermal activity data was unusable.

**Table 1:** Socio-demographic characteristics.

	Children (n = 83)		Parents (n = 73)	
	Mean	SD	Mean	SD
Age	9.7	1.4	41.1	4.8
Sex (M:F)	40:43	N/A	32:41	N/A
Education (years)	4.7	1.4	15.7	2.7
Shock intensity level (mA)	N/A	N/A	2.2	1.4

F, female; M, male; mA, milliamperes; SD, Standard deviation.

### Parents

#### Fear conditioning

During the direct fear conditioning phase, parents’ SCRs were larger for the conditioned stimulus paired to a shock (CS +) than the one that was not (CS −) [F(1,71) = 45.37, *p* < 0.001, d = 0.53], which confirmed successful fear conditioning. There was no main effect or interaction involving Parent’s sex. Importantly, the level of shock chosen by the parent was not associated with his/her SCR during the fear conditioning phase [r(72) = − 0.155, *p* = 0.192].

### Children

#### Observational fear learning

During the observational fear learning session, children’s SCRs were significantly larger for CS + than CS − [F(1,76) = 11.71, *p* = 0.001, d = 0.33]. There were no other main effect or interaction (all F’s < 2.6, all *p* values > 0.1).

#### Direct expression test, extinction learning, and direct retention test

**Figure 2:**
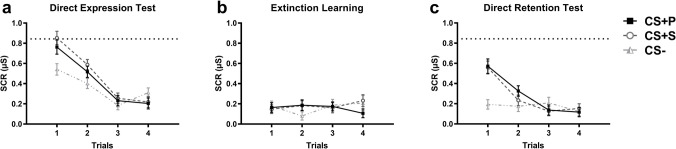
Skin conductance responses for the direct expression test, extinction learning, and direct retention test in children. (**a**) After seeing their parent as well as a stranger receiving shock for the CS + , children presented higher SCRs for both CS + s, in comparison to the CS − (direct expression test). (**b**) At the end of that phase, the fear responses to all three CSs have extinguished (extinction learning). (**c**) The day after (direct retention test), children exhibited higher SCRs for both CS + s than the CS −. In each panel, black lines represent CS + Parent, dashed dark grey lines represent CS + Stranger, and dashed light grey lines represent CS −. Error bars are standard error of the mean. The dashed horizontal line indicates the highest level of fear (SCR) expressed during the direct expression test. CS + P = CS + Parent; CS + S = CS + Stranger; µS = microsiemens.

During testing phases, the ANOVA revealed a significant Phase by Stimulus by Trial interaction [F(9.64,723.26) = 2.48, *p* = 0.007, ηp^2^ = 0.032]. No interaction involving parents’ or children’s sex were found [all F’s < 2.03, all *p* values > 0.09]. The Phase by Stimulus by Trial interaction was decomposed for each phase. There were a Stimulus by Trial interaction for both the direct expression test [F(6,450) = 4.79, *p* < 0.001, ηp^2^ = 0.060] and the direct retention test (Day 2) [F(4.88,366.11) = 6.15, *p* < 0.001, ηp^2^ = 0.076] phases, but not during the fear extinction learning phase [F(4.79,359.19) = 1.31, *p* 0.262, ηp^2^ = 0.017] (Fig. 2). The latter result suggests that the three stimuli elicited similar physiological reactions during that phase. During the direct expression test, significant differentiation between stimuli was found for the first [F(2,156) = 10.84, *p* < 0.001, ηp^2^ = 0.122] and the second [F(2,156) = 6.50, *p* = 0.002, ηp^2^ = 0.077] trials. For the first trial, both the CS + Parent [*p* = 0.007, d = 0.40] and the CS + Stranger [*p* < 0.001, d = 0.57] differed from the CS −. For the second trial, only the CS + Stranger significantly differed from the CS − [*p* = 0.004, d = 0.45]. On Day 2 (direct retention test), significant differentiation between stimuli was found for the first trial [F(2,156) = 16.85, *p* < 0.001, ηp^2^ = 0.178]. Post-hoc Bonferroni tests revealed that both the CS + Parent [*p* < 0.001, d = 0.71] and the CS + Stranger [*p* < 0.001, d = 0.73] differed from the CS − on this trial.

#### Physiological synchrony

**Figure 3:**
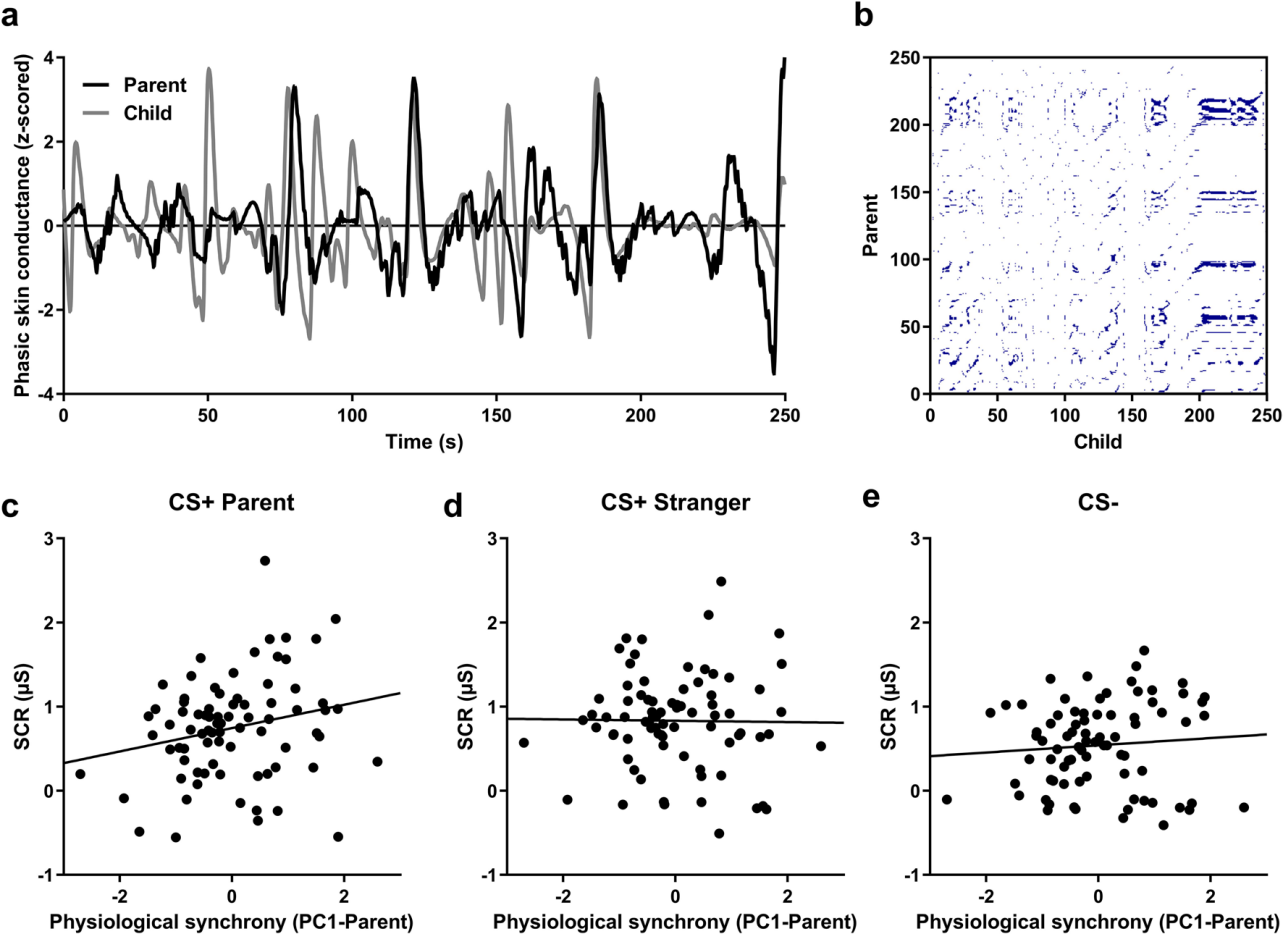
Parent–child’s physiological synchrony predicts fear learning in children, only for the parent cue. (**a**) Example of the z-scored phasic skin conductance for a parent and his/her child during the whole observational learning stage. (**b**) Cross-recurrence plot obtained from the same parent–child dyad. (**c**) Physiological synchrony between children and parents during the observational learning stage successfully predicted the fear response in children for the first CS + Parent presentation during direct expression test. Physiological synchrony between children and parents did not predict the fear response for neither the CS + Stranger (**d**), nor the CS − (**e**).

In child-parent dyads, the first factor of the principal component analysis (PCA) (PC1-Parent) conducted on determinism (DET), laminarity (LAM), length of the longest diagonal (MaxL), and relative entropy (rENTR) measures obtained while children watched the video of their parent explained 65% of the variance. We first tested whether this factor (PC1-Parent) correlated with the shock level chosen by the participant. This correlation did not reach significance [r(76) = − 0.195, *p* = 0.092], but the detected trend suggested a potential relationship between the shock intensity and the parent–child synchrony. The PC1-Parent factor predicted the conditioned response to the first CS + Parent presentation during the direct expression test [r(76) = 0.228, *p* = 0.047]. It did not predict the conditioned response to the first CS + Stranger presentation [r(76) = − 0.017, *p* = 0.881], nor the first CS − presentation [r(76) = 0.056, *p* = 0.632] (see Fig. 3), suggesting that greater physiological synchrony within the dyad predicts greater fear responses specifically to the danger-related cue specific to the parent. There was a significant difference between the strength of the correlation between the PC1-Parent and SCRs to the first CS + Parent presentation (r = 0.228) and the strength of the correlation between the PC1-Parent and SCRs to the first CS + Stranger presentation (r = − 0.017) [z = 2.00, *p* = 0.045]. There was no other significant difference between these correlation coefficients [all z-values < 1.4, all *p* values > 0.1].

**Figure 4:**
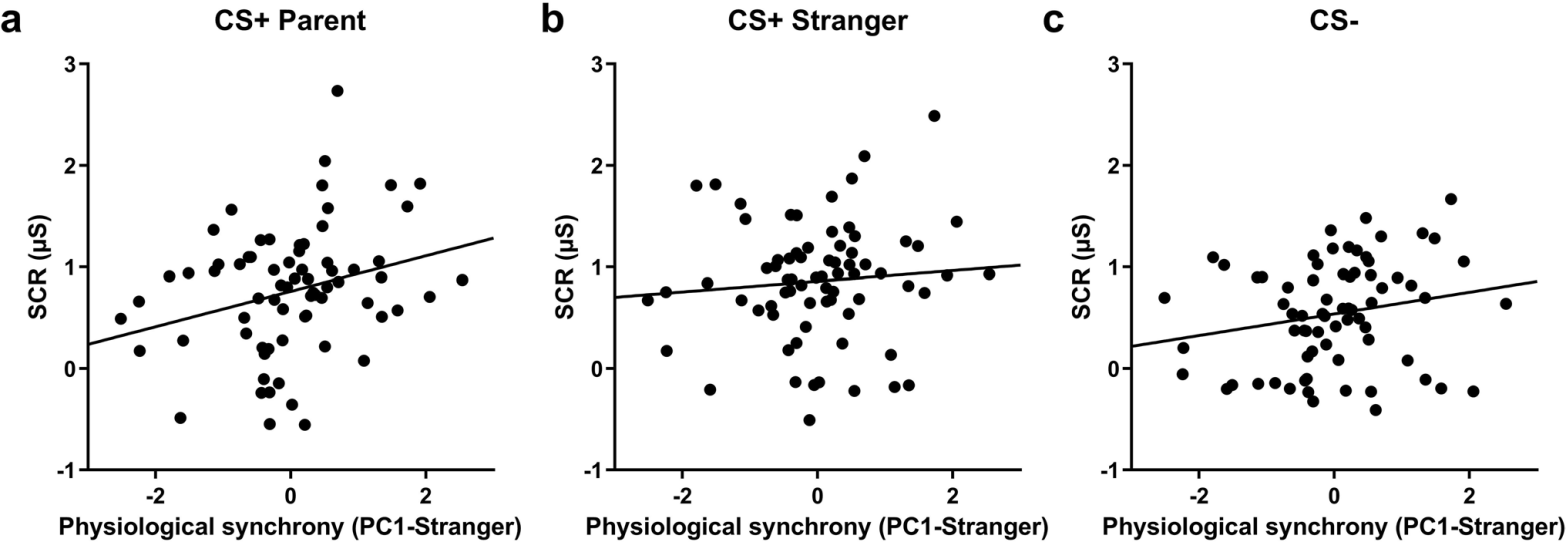
Stranger-child’s physiological synchrony predicts fear learning in children, only for the parent cue. (**a**) Physiological synchrony between children and strangers during the observational learning stage successfully predicted the fear response in children for the first CS + Parent presentation during the direct expression test. Physiological synchrony between children and strangers did not predict the fear response for neither the CS + Stranger (**b**), nor the CS − (**c**).

We also calculated the synchrony between strangers’ electrodermal activity while undergoing fear conditioning and children’s electrodermal activity while watching the video of the stranger. Here also, we conducted a PCA on DET, LAM, MaxL, and rENTR. The first factor of this PCA (PC1-Stranger) explained 63% of the variance, and only predicted the conditioned response to the first CS + Parent presentation [r(71) = 0.281, *p* = 0.018]. It did not predict the conditioned response to the first CS + Stranger presentation [r(71) = 0.090, *p* = 0.456], but tended to predict the first CS − presentation [r(71) = 0.205, *p* = 0.086] (see Fig. 4). These three correlation coefficients did not differ from one another [all z-values < 1.5, all *p* values > 0.1]. Also, there was no significant difference between the strength of the correlation between the PC1-Parent and SCRs to the first CS + Parent presentation (r = 0.228) and the strength of the correlation between the PC1-Stranger and SCRs to the first CS + Stranger presentation (r = 0.090) [z = 0.89, *p* = 0.37].

#### Subjective reports of contingency learning

We tested whether children learned the contingency between the conditioned stimulus (CS) and the unconditioned stimulus (US) (CS-US contingency). This was done by asking children to correctly identify which color was associated with the shock for the parent and which color was associated with the shock for the stranger. From the 83 children included in the study, 64 explicitly learned the CS-US contingency whereas 19 children failed to acquire the CS-US contingency. We assessed whether those two groups differed in terms of age and sex distribution. Children among the group who learned the CS-US contingency tended to be older than those who did not [t(81) = − 1.79, *p* = 0.077, d = 1.13]. There was, however, no difference in sex distribution between groups [χ^2^(1, n = 83) = 1.27, *p* = 0.259].

All the previous analyses were repeated for children who learned the CS-US contingency and for those who did not learn the CS-US contingency. Overall, the effects reported above remained in the group who learned the contingency. However, children who did not learn the contingency exhibited similar levels of fear to all CSs (CS + Parent, CS + Stranger, and CS −). For statistical details, see Supplementary information.

## Discussion

This study aimed to assess the physiological correlates of observational fear learning among parent–child dyads. Children watched two videos: one of their parent receiving shocks for a given cue (CS + Parent) and no shock for another cue (CS −), and one of a stranger receiving shocks for another cue (CS + Stranger) and where the CS − was the same. Children were then exposed to these cues without receiving any shocks. We showed for the first time that children acquire differential fear conditioning vicariously as indexed by SCR, and that familiarity with the demonstrator (parent or a stranger) does not impact their conditioned fear responses. Lastly, our results suggest that the more physiologically synchronized the parent–child dyad is, the higher the fear responses expressed by the child when later exposed to the CS + Parent.

Our results support prior studies that had shown that children’s self-reported fear levels could be modulated by pairing a given animal with a fearful facial expression of their mother or a stranger or by instructions received from an adult^6,23,24^. Our study provides psychophysiological measures that go beyond the subject’s self-report and demonstrates that the discrimination between fearful and safety signals is not only measurable by self-report measures, but also through physiological measures. The fact that children in our study exhibited similar SCR to the CS + Parent and the CS + Stranger suggests that, on average, children can learn fear equivalently from a parent and a stranger. This suggests that in our sample, some children learned more from the stranger than they did from their parent and vice-versa. Although this finding is aligned with previous studies on observational fear learning in children that have used subjective fear levels, it nonetheless underscores the importance of assessing variables that could explain better learning from a familiar adult (in this case, the parent) or a stranger. Our sample was composed of healthy children learning from healthy adults. It will be interesting in the future to assess how children learn from a parent or from a stranger exhibiting exaggerated fear levels. Moreover, other factors that are specific to the individuals or the dyads should be further studied, such as the attachment relationship or the observer’s level of empathy. As an example, Callaghan and colleagues^25^ found that the presentation of parent-related visual cues induced lower amygdala reactivity in children, but that effect was not observed in children who previously experienced institutional care^25^. This result highlights the importance of further considering the role of interindividual and inter-dyadic factors that could modulate fear learning.

One of the novel and most exciting findings of our study is that the synchrony between the parent’s SCR during conditioning and their child during the observational learning phase was predictive of the child’s physiological reactivity when directly presented with the CS + Parent (direct expression test). These data are consistent with a prior study^26^ that demonstrated a similar association among adults performing an observational fear learning. As argued by Pärnamets and colleagues, synchrony might reflect the observer mirroring the demonstrator’s autonomic activity, which would in turn facilitate fear learning for the observer. In our sample, synchrony between the parent and the child was correlated with the child’s SCR to the CS + Parent, but not for the CS + Stranger and the CS −, suggesting specificity to the fear-related stimulus for the parent. Further support for this argument comes from the fact that the synchrony between the stranger and the child did not predict the child’s SCR to the CS + Stranger. This finding however needs to be interpreted cautiously for two main reasons. First, although not significant, this correlation did not differ statistically from the significant association found between parent–child synchrony and the child’s SCR to the CS + Parent. Second, the fact that all children who came with their father saw the same stranger man and all children who came with their mother saw the same stranger woman could also bias some of the stranger-child synchrony findings, as all variability comes from the child and the demonstrator does not contribute any variability. This might also explain the fact that we have found a significant association between the stranger–child synchrony and the child’s SCR to the CS + Parent. Taken together, these findings suggest that the propension of a child to synchronize his/her physiological state with the demonstrator might hold across contexts and render some children more receptive to observational learning. Nonetheless, our data highlight the importance of further considering physiological synchrony, as this may be a mechanism contributing to observational fear learning within families. Higher synchrony between a parent and a child could serve as an adaptive function. For example, by promoting more efficient learning, a stronger synchrony could help in adapting to the environment by having a better understanding of what is dangerous and what is safe. This being said, whether such synchrony always serves as an advantage remains to be tested. For example, if a child learns from a parent suffering from a fear or anxiety-related disorder, a higher synchrony could promote transmission of this maladaptive fear. In other words, risk for anxiety disorders may be preferentially transmitted from parent to child only in cases with high physiological synchrony. It is therefore important to examine individual and dyadic related factors that could modulate the strength of the synchrony.

Apart from tracking observational fear learning, our study allowed to examine whether the fear memory would undergo extinction and to test for delayed retention of fear on the next day. At the end of Day 1, children exhibited low SCR levels for all three CSs, suggesting successful extinction learning for both the CS + Parent and the CS + Stranger. On the following day, when children were tested for direct retention, there was some spontaneous recovery of fear for both CS + s relative to the CS −. Although some savings of extinction from session 1 could be observed, elevated SCR to the CS + compared to the CS − suggest a significant return of fear, which is distinct from prior studies in adult populations using direct fear conditioning paradigms^27,28^. We speculate that the significant spontaneous recovery we observed might be related to distinct neural mechanisms mediating fear extinction learning and recall within this age group compared to adults. It has been shown that extinction learning might be more dependent on the amygdala in early development and might recruit more the hippocampus and the medial prefrontal cortex (mPFC) during later stages of development^29^. Human studies have also reported that the functional and the structural connectivity between the amygdala and the prefrontal cortex is not mature yet in childhood^30–32^. Given the inhibitory role of the prefrontal cortex on the amygdala and its implication in extinction learning and retention, the young age of our sample could account for the important return of fear to the CS + during extinction recall. Adding a neuroimaging component to this protocol would allow testing this hypothesis. Importantly, a recent observational fear learning study performed in adults also reported elevated SCR to the CS + after a delay^33^. It is therefore possible that the nature of the protocol (vicarious learning vs. direct learning) might contribute to a stronger return of fear after a delay. Future studies should therefore test whether fear learning that occurs through observation is more resistant to extinction.

In the same vein, previous studies using direct fear conditioning protocols have shown that brain structures involved in fear learning and regulation, such as the amygdala, the hippocampus and the ventromedial prefrontal cortex, change throughout development^29,34^. Our study and its design could set the stage for future important mechanistic and clinical studies in this domain. For example, implementing this protocol in an fMRI scanner would provide valuable information on how fears are learned in children when observing their parent. Neural correlates of observational fear learning could also inform us about individual differences promoting risk and resilience, for example in families where parents suffer from pathologies characterized by dysregulated fear levels.

While novel and original, our study has some limitations. For example, it would have been interesting to complement the physiological recordings with subjective fear measures. This would have allowed assessing whether subjective and physiological fear levels correlate and under which conditions the two are related (or not). While our sample size was similar to other fear conditioning studies^4,6,7,35–37^, a larger sample would allow investigating sex differences in children as well as sex congruence within dyads. Given the important sex differences in anxiety and fear-related disorders^38–42^, it is crucial to study both girls and boys and to assess effects of sex congruence with their parents. Moreover, trait empathy has been shown to contribute to observational fear learning^43^. Having such measure in our study could have helped understanding differences individual differences with regards to observational fear leaning in children.

In sum, our novel protocol and experimental design enabled us to gather physiological data to study not only fear acquisition but also fear regulation, through the addition of extinction learning and retention test. Moreover, through the acquisition of physiological data from both the parent and the child, the protocol allowed us to examine synchronization within the dyad to better disentangle the mechanisms underlying observational fear learning in children. Future studies will allow investigating the individual and environmental factors that modulate one’s vulnerability to observational fear learning in the context of the familial environment, especially in at-risk families where the parent suffers from anxiety or fear-related psychopathologies.

## Methods

### Participants

Eighty-three healthy parent–child dyads were recruited through announcements on social media and posters in the surroundings of the research center. These dyads consisted of 73 parents and 83 children (10 parents participated with two of their children). There were 19 father–son dyads, 17 father–daughter dyads, 21 mother–son dyads, and 26 mother–daughter dyads. Inclusion criteria for participation in this study were (i) that the child was aged between 8 and 12 years old and accompanied by one of his/her parents; (ii) that the child and the parent understood and spoke French; (iii) and that both had normal or corrected vision. Exclusion criteria for children were (i) history of mental illness, brain damage, or significant developmental delay; (ii) an unstable or severe medical condition; and (iii) current or past use of psychiatric medication. Exclusion criteria for the parents were (i) history of bipolar, psychotic, or substance use disorder; (ii) an unstable or severe medical condition; and (iii) pregnancy for the mother. This project was approved by the institutional review board of the *CIUSSS-de-l’Est-de-l’Île-de-Montréal* and was conducted in conformity with the Declaration of Helsinki. Informed consent was obtained from all subjects, where parents gave informed consent for themselves and their child. Written consent was obtained from all parents and written assent was obtained from all children.

### Procedures

Parent–child dyads took part in a 2-day fear conditioning and extinction paradigm (see Fig. 1). Of note, informed consent from people depicted in Fig. 1 was obtained to publish the images, and the stranger man was not a study participant.

#### Direct fear conditioning protocol for the parent

On Day 1, we attached two Ag/AgCl electrodes to the palm of the parent’s left hand. Electrical shocks were delivered through two electrodes placed on the index and middle fingers of the parent’s right hand. Parents were then instructed to choose a level of shock that was annoying but not painful. Parents underwent the habituation phase, where two stimuli (colored lamps) were presented without any shock. This was followed by the direct fear conditioning phase, with 8 presentations of the conditioned stimulus (CS + Parent; blue or red lamp) and 4 presentations of the CS − (yellow lamp). Shocks (500 ms) were delivered for 5 of the 8 CS + presentations. We recorded a video of the parent during the conditioning phase that captured the stimuli being presented and the parent’s facial and bodily reactions. Another video was recorded prior to the start of the project. That video consisted of a stranger adult going through the fear conditioning protocol. The stranger adult was exposed to the same protocol than the parent except that a different colored lamp was used for the CS + (e.g., blue or red lamp, CS + Stranger). The CS − for the parent and the stranger was the same colored lamp. The video of the stranger was standardized across all participants according to the parent’s sex (children participating with their mother saw the same video of a stranger woman and children participating with their father saw the same video of a stranger man). Both stranger adults were Caucasians.

#### Observational fear learning for the child

On Day 1, we attached two Ag/AgCl electrodes to the palm of the child’s left hand. Children were then exposed to a habituation phase during which they saw all three stimuli (i.e., colored lamps), none of which were paired with a shock. Afterwards, the child was presented with both videos without audio (observational learning stage). The order of the video presentations was counterbalanced across participants. After each video, we asked the child which colors were presented, whether shocks were delivered or not, and if so, to which color(s) shocks were associated. This was used to assess contingency learning. Then, shock electrodes were placed on the index and middle finger of the child’s dominant hand. The child was told that he/she would be presented with the colored lamps directly and that he/she might receive a shock. We instructed the child that if a shock was delivered, it would be at a level considered by same-age children to be annoying but not painful. No shocks were delivered to the child at any point during the study. The child was then presented with all three cues (CS + Parent, CS + Stranger, and CS −), 8 times each. The first four trials of that session served to examine whether fear was learned (direct expression test) and the last four trials of that session served to examine fear extinction learning (given that no shocks were delivered, extinction learning eventually took place).

The next day, skin conductance electrodes and shock electrodes were once again placed on the child (see procedure of Day 1). Direct retention was tested by presenting directly all three CSs, 8 times each. At the end of the experiment, there was a debriefing session with the child and the parent. They were explained why deception was used and why maintaining uncertainty about shock delivery was necessary to induce conditioned fear responses.

### Psychophysiological recordings and data processing

#### Skin conductance recordings

Skin conductance levels (SCL) were recorded continuously during all phases of the paradigm (MP160 recording system, Biopac Systems Inc., Goleta, CA). Skin conductance responses (SCRs) were obtained by subtracting the mean SCL during the 2-s interval prior to CS onset from the maximum SCL (peak) obtained during the 6-s interval following CS onset. SCR data were then square-root transformed.

#### Physiological synchrony

Physiological synchrony analyses were performed for the conditioning session (when parents and strangers underwent direct conditioning and when the children viewed both videos, i.e., observational learning stage). The raw electrodermal activity signal recorded during this session was filtered with a 0.05–1 Hz passband and downsampled to 8 Hz. This signal was z-scored to ensure comparability between parents and children as well as between strangers and children.

Here, we used cross-recurrence quantification analyses (CRQA) as a proxy for physiological synchrony. These analyses were performed in R with the crqa package^44^. Basically, CRQA is used to quantify the time-dependent coupling of two signals^45^. This analysis yields a cross-recurrence plot (see Fig. 3b for an example), where blue dots represent states of recurrence between two-time series. To be considered as recurrent, points must be within a given radius.

Using the optimizeParam function, parameters (radius, delay, embed dimension) were set individually for each pair to provide a mean recurrence rate between 2 and 4%^26^. The recurrence rate corresponds to the percentage of points in a cross-recurrence plot forming recurrence. A cross-recurrence plot was therefore obtained for each pair (parent–child, stranger-child). Synchrony metrics were extracted from each of these cross-recurrence plots. We used the same CRQA metrics as Pärnamets and colleagues^26^ for our analyses: determinism (DET), laminarity (LAM), length of the longest diagonal (MaxL), and relative entropy (rENTR). DET and LAM represent the percentage of recurrence points forming diagonal and vertical lines, respectively. MaxL represents the length of the longest diagonal in the cross-recurrence plot. rENTR represents the Shannon entropy of the diagonal lines in the cross-recurrence plot, normalized by the number of lines^44^.

From the 79 parent–child dyads included in previous analyses, two were excluded from synchrony analyses, since it was not possible to optimize their parameters to obtain a recurrence rate between 2 and 4%. Another parent–child dyad was excluded because of poor data quality for the parent. Physiological synchrony analyses were therefore performed on 76 parent–child dyads. Eight children were excluded from stranger-child synchrony analyses, because of data acquisition problems during the viewing of the stranger’s video. Physiological synchrony between strangers’ and children’s electrodermal activity was therefore computed for 71 children. For both parent–child and stranger-child analyses, we performed a principal component analysis (PCA) using varimax-rotation on these four metrics to reduce the number of analyses^26,46^. We used the resulting first factors (PC1 Parent–child, PC1 Stranger-child) of this analysis to predict children’s CS + and CS − responses during the direct expression test.

### Data analysis

To assess fear conditioning in parents, SCRs were analyzed with a mixed ANOVA with the within-subjects factor Stimulus (CS + , CS −) and the between-subjects factor Parent’s sex (male, female). In children, SCRs during the observational learning phase were analyzed with a mixed ANOVA, with the within-subjects factors Stimulus (CS + , CS −) and Demonstrator (parent, stranger), and the between-subjects factors Demonstrator’s sex and Child’s sex (male, female). In order to test the direct expression of fear, fear extinction learning, and the direct retention of fear within the same statistical model, we only included the first four trials of the direct retention test, so that all phases have four trials. Data from these three phases were assessed with a mixed ANOVA, with the within-subjects factors Phase (direct expression test, fear extinction learning, direct retention test), Stimulus (CS + Parent, CS + Stranger, CS −) and Trials (4 trials), and the between-subjects factors Parent’s sex and Child’s sex (male, female). Significant interactions were decomposed with subsequent ANOVAs and post-hoc Bonferroni tests. Greenhouse–Geisser corrections were applied when sphericity assumption was not met. To assess the role of physiological synchrony in fear learning, we conducted Pearson correlations between both the Parents’ and Strangers’ PC1 and children’s SCRs for the first trial of each stimulus type during the direct expression test. To compare correlations coefficients between conditions, we used the procedure of Hittner et al.^47^, which is a modification of Dunn and Clark^48^ using a backtransformed average Fisher Z procedure^49^. This test is implemented in the cocor (https://comparingcorrelations.org) online software^50^.

## Supplementary information

Supplementary Information.

## Data Availability

The datasets generated and analyzed during the current study are available from the corresponding author on reasonable request.

## References

[1] Dias BG, Ressler KJ (2014). Parental olfactory experience influences behavior and neural structure in subsequent generations. Nat. Neurosci..

[2] Debiec J, Sullivan RM (2014). Intergenerational transmission of emotional trauma through amygdala-dependent mother-to-infant transfer of specific fear. Proc. Natl. Acad. Sci. U. S. A..

[3] Bandura A (1977). Social Learning Theory.

[4] Askew C, Field AP (2008). The vicarious learning pathway to fear 40 years on. Clin. Psychol. Rev..

[5] Mineka S, Cook M (1993). Mechanisms involved in the observational conditioning of fear. J. Exp. Psychol. Gen..

[6] Dunne G, Askew C (2013). Vicarious learning and unlearning of fear in childhood via mother and stranger models. Emotion.

[7] Waters AM, Henry J, Neumann DL (2009). Aversive Pavlovian conditioning in childhood anxiety disorders: impaired response inhibition and resistance to extinction. J. Abnorm. Psychol..

[8] Reynolds G, Field AP, Askew C (2014). Effect of vicarious fear learning on children’s heart rate responses and attentional bias for novel animals. Emot. Wash. DC.

[9] Reynolds G, Field AP, Askew C (2018). Reductions in children’s vicariously learnt avoidance and heart rate responses using positive modeling. J. Clin. Child Adolesc. Psychol..

[10] Vervliet B, Craske MG, Hermans D (2013). Fear extinction and relapse: state of the art. Annu. Rev. Clin. Psychol..

[11] Kessler RC (2005). Lifetime prevalence and age-of-onset distributions of DSM-IV disorders in the National Comorbidity Survey Replication. Arch. Gen. Psychiatry.

[12] Cohen P (1993). An epidemiological study of disorders in late childhood and adolescence–I. Age- and gender-specific prevalence. J. Child Psychol. Psychiatry.

[13] Beesdo K, Knappe S, Pine DS (2009). Anxiety and anxiety disorders in children and adolescents: developmental issues and implications for DSM-V. Psychiatr. Clin. North Am..

[14] Ost LG (1987). Age of onset in different phobias. J. Abnorm. Psychol..

[15] Eisenberg N, Cumberland A, Spinrad TL (1998). Parental socialization of emotion. Psychol. Inq..

[16] Gunnar MR, Hostinar CE, Sanchez MM, Tottenham N, Sullivan RM (2015). Parental buffering of fear and stress neurobiology: reviewing parallels across rodent, monkey, and human models. Soc. Neurosci..

[17] Winnicott DW (1960). The theory of the parent-infant relationship. Int. J. Psychoanal..

[18] Callaghan B (2019). Using a developmental ecology framework to align fear neurobiology across species. Annu. Rev. Clin. Psychol..

[19] Lieb R (2000). Parental psychopathology, parenting styles, and the risk of social phobia in offspring: a prospective-longitudinal community study. Arch. Gen. Psychiatry.

[20] Mancini C, van Ameringen M, Szatmari P, Fugere C, Boyle M (1996). A high-risk pilot study of the children of adults with social phobia. J. Am. Acad. Child Adolesc. Psychiatry.

[21] Poole KL, Van Lieshout RJ, McHolm AE, Cunningham CE, Schmidt LA (2018). Trajectories of social anxiety in children: influence of child cortisol reactivity and parental social anxiety. J. Abnorm. Child Psychol..

[22] Schechter DS (2017). Maternal PTSD and corresponding neural activity mediate effects of child exposure to violence on child PTSD symptoms. PLoS ONE.

[23] Askew C, Field AP (2007). Vicarious learning and the development of fears in childhood. Behav. Res. Ther..

[24] Field AP, Argyris NG, Knowles KA (2001). Who’s afraid of the big bad wolf: a prospective paradigm to test Rachman’s indirect pathways in children. Behav. Res. Ther..

[25] Callaghan BL (2019). Decreased amygdala reactivity to parent cues protects against anxiety following early adversity: an examination across 3 years. Biol. Psychiatry Cogn. Neurosci. Neuroimaging.

[26] Pärnamets P, Espinosa L, Olsson A (2020). Physiological synchrony predicts observational threat learning in humans. Proc. R. Soc. B Biol. Sci..

[27] Milad MR (2007). Recall of fear extinction in humans activates the ventromedial prefrontal cortex and hippocampus in concert. Biol. Psychiatry.

[28] Zeidan MA (2012). Test-retest reliability during fear acquisition and fear extinction in humans. CNS Neurosci. Ther..

[29] Shechner T, Hong M, Britton JC, Pine DS, Fox NA (2014). Fear conditioning and extinction across development: evidence from human studies and animal models. Biol. Psychol..

[30] Swartz JR, Carrasco M, Wiggins JL, Thomason ME, Monk CS (2014). Age-related changes in the structure and function of prefrontal cortex-amygdala circuitry in children and adolescents: a multi-modal imaging approach. NeuroImage.

[31] Lebel C (2012). Diffusion tensor imaging of white matter tract evolution over the lifespan. NeuroImage.

[32] Gabard-Durnam LJ (2014). The development of human amygdala functional connectivity at rest from 4 to 23 years: a cross-sectional study. NeuroImage.

[33] Esser R, Fuss J, Haaker J (2020). Initial evidence for pharmacological modulation of observational threat learning by the GABAergic, but not the noradrenergic system in humans. Behav. Res. Ther..

[34] Lau JY (2011). Distinct neural signatures of threat learning in adolescents and adults. Proc. Natl. Acad. Sci..

[35] Jovanovic T (2014). Development of fear acquisition and extinction in children: effects of age and anxiety. Neurobiol. Learn. Mem..

[36] Waters AM, Peters R-M, Forrest KE, Zimmer-Gembeck M (2014). Fear acquisition and extinction in offspring of mothers with anxiety and depressive disorders. Dev. Cogn. Neurosci..

[37] Egliston K-A, Rapee RM (2007). Inhibition of fear acquisition in toddlers following positive modelling by their mothers. Behav. Res. Ther..

[38] McLean CP, Asnaani A, Litz BT, Hofmann SG (2011). Gender differences in anxiety disorders: prevalence, course of illness, comorbidity and burden of illness. J. Psychiatr. Res..

[39] McLean CP, Anderson ER (2009). Brave men and timid women? A review of the gender differences in fear and anxiety. Clin. Psychol. Rev..

[40] Lewinsohn PM, Gotlib IH, Lewinsohn M, Seeley JR, Allen NB (1998). Gender differences in anxiety disorders and anxiety symptoms in adolescents. J. Abnorm. Psychol..

[41] Rapee RM, Schniering CA, Hudson JL (2009). Anxiety disorders during childhood and adolescence: origins and treatment. Annu. Rev. Clin. Psychol..

[42] Roza SJ, Hofstra MB, van der Ende J, Verhulst FC (2003). Stable prediction of mood and anxiety disorders based on behavioral and emotional problems in childhood: a 14-year follow-up during childhood, adolescence, and young adulthood. Am. J. Psychiatry.

[43] Kleberg JL, Selbing I, Lundqvist D, Hofvander B, Olsson A (2015). Spontaneous eye movements and trait empathy predict vicarious learning of fear. Int. J. Psychophysiol..

[44] Coco MI, Dale R (2014). Cross-recurrence quantification analysis of categorical and continuous time series: an R package. Front. Psychol..

[45] Wallot S, Leonardi G (2018). Deriving inferential statistics from recurrence plots: a recurrence-based test of differences between sample distributions and its comparison to the two-sample Kolmogorov-Smirnov test. Chaos Interdiscip. J. Nonlinear Sci..

[46] Mønster D, Håkonsson DD, Eskildsen JK, Wallot S (2016). Physiological evidence of interpersonal dynamics in a cooperative production task. Physiol. Behav..

[47] Hittner JB, May K, Silver NC (2004). Testing dependent correlations with nonoverlapping variables: a Monte Carlo simulation. J. Exp. Educ..

[48] Dunn OJ, Clark V (1971). Comparison of tests of the equality of dependent correlation coefficients. J. Am. Stat. Assoc..

[49] Fisher, R. A. On the probable error of a coefficient of correlation deduced from a small sample. *Metron* **1**, 3–32 (1921).

[50] Diedenhofen B, Musch J (2015). cocor: a comprehensive solution for the statistical comparison of correlations. PLoS ONE.

