# Supplementary material for: Vicarious conditioned fear acquisition and extinction in child–parent dyads
Source: Sci Rep. 2020 Oct 13;10:17130. doi: 10.1038/s41598-020-74170-1 (PMC7555483; doi:10.1038/s41598-020-74170-1)
Supplement: Supplementary file 1 — Supplementary Information. [file 41598_2020_74170_MOESM1_ESM.docx]

**Supplementary Information**

**Vicarious conditioned fear acquisition and extinction**

**in child-parent dyads**

Marie-France Marin^1,2,*^, Alexe Bilodeau-Houle^1,2,3^, Simon Morand-Beaulieu^2,4,7^, Alexandra Brouillard^1,2,3^, Ryan J. Herringa^5^, Mohammed R. Milad^6^

^1^ Department of Psychology, Université du Québec à Montréal, 100 Sherbrooke West Street, Montreal, QC H2X 3P2, Canada

^2^ Research Center of the Institut universitaire en santé mentale de Montréal, 7331 Hochelaga Street, Montreal, QC H1N 3V2, Canada

^3^ Department of Psychology, Université de Montréal, 2900 Edouard-Montpetit Blvd, Montreal, QC H3T 1J4, Canada

^4^ Department of Neurosciences, Université de Montréal, 2900 Edouard-Montpetit Blvd, Montréal, QC H3T 1J4, Canada

^5^ Department of Psychiatry, University of Wisconsin School of Medicine and Public Health, 750 Highland Ave, Madison, WI 53726, USA

^6^ Department of Psychiatry, New York University Grossman School of Medicine, 530 1st Ave, New York, NY 10016, USA

^7^ Currently with the Child Study Center, Yale University School of Medicine, 230 S Frontage Rd, New Haven, CT 06519, USA

*Corresponding author's email: [marin.marie-france@uqam.ca](mailto:marin.marie-france@uqam.ca)

**Supplementary results**

**For children who understood contingency**

Observational fear learning

Stimulus main effect: [F(1,57) = 17.21, p < .001, d = .49]

Direct expression test, extinction learning, and direct retention test

Phase by Stimulus by Trial interaction: [F(8.61,481.95) = 2.14, p = .013, ηp² = .037]

Stimulus by Trial interaction:

Direct expression test: [F(6,336) = 4.89, p < .001, ηp² = .080]

1^st^ trial, Stimulus main effect: [F(2,112) = 10.16, p < .001, ηp² = .154]

Bonferroni: CS+Parent > CS-: [p = .007, d = .50]

CS+Stranger > CS-: [p < .001, d = .64]

2^nd^ trial, Stimulus main effect: [F(2,112) = 7.51, p = .001, ηp² = .118]

Bonferroni: CS+Parent > CS-: [p = .033, d = .42]

CS+Stranger > CS-: [p = .002, d = .54]

Fear extinction learning: [F(4.43,247.89) = 0.61, p = .673, ηp² = .011]

Direct retention test (Day 2): [F(4.34,242.92) = 4.70, p = .001, ηp² = .077]

1^st^ trial, Stimulus main effect: [F(2,112) = 14.34, p < .001, ηp² = .204]

Bonferroni: CS+Parent > CS-: [p < .001, d = .78]

CS+Stranger > CS-: [p < .001, d = .85]

Physiological synchrony between children and parents

Correlation between PC1 and first CS+P [r(57) = .241, p = .070], CS+S [(57) = .031, p = .817], CS- [r(57) = .064, p = .634].

Physiological synchrony between children and strangers

Correlation between PC1 and first CS+P [r(53) = .283, p = .040], CS+S [r(53) = .041, p = .773], CS- [r(53) = .178, p = .202]

**For children who did not understand contingency**

Direct expression test

No stimulus main effect: [F(1,15) = .10, p = .752, d = .07]

Fear extinction learning

No stimulus main effect: [F(2,30) = 1.21, p = .312, ηp² = .075].

Direct retention test (Day 2)

No stimulus main effect: [F(1.39,19.47) = 1.11, p = .329, ηp² = .073].
